# Validation of visual analog scales of mood and anxiety at the workplace

**DOI:** 10.1371/journal.pone.0316159

**Published:** 2024-12-31

**Authors:** Frédéric Dutheil, Clara Palgen, Georges Brousse, Thomas Cornet, Martial Mermillod, Ines Lakbar, Guillaume Vallet, Julien S. Baker, Jeannot Schmidt, Barbara Charbotel, Bruno Pereira, Louis Delamarre

**Affiliations:** 1 University Hospital of Clermont–Ferrand, CHU Clermont–Ferrand, Preventive and Occupational Medicine, Université Clermont Auvergne, Clermont–Ferrand, France; 2 NPSY-SYDO, University Hospital of Clermont–Ferrand, CHU Clermont–Ferrand, Psychiatry, Université Clermont Auvergne, Clermont-Ferrand, France; 3 WittyFit, Paris, France; 4 CNRS, LPNC, Univ. Grenoble Alpes, Grenoble, France; 5 Institut Universitaire de France, Paris, France; 6 Intensive Care Unit, Anesthesia and Critical Care Department (DAR-B), Saint-Eloi Teaching Hospital, University of Montpellier, Montpellier, France; 7 CNRS, LaPSCo, Physiological and Psychosocial Stress, Université Clermont Auvergne, Clermont-Ferrand, France; 8 Centre for Health and Exercise Science Research, Department of Sport, Physical Education and Health, Hong Kong Baptist University, Kowloon Tong, Hong Kong; 9 CNRS, LaPSCo, Physiological and Psychosocial Stress, University Hospital of Clermont-Ferrand (CHU), Emergency Department, Université Clermont Auvergne, Clermont-Ferrand, France; 10 Univ Lyon 1, IFSTTAR, UMRESTTE, UMR_T9405, University of Lyon, Lyon, France; 11 University Hospital of Clermont Ferrand, CHU Clermont–Ferrand, Clinical Research and Innovation Direction, Clermont-Ferrand, France; Telematic University San Raffaele Rome: Universita Telematica San Raffaele Roma, ITALY

## Abstract

The Hospital Anxiety and Depression Scale (HADS) is commonly used to detect depressive or anxious states, but its 14-item questionnaire is time-consuming. Visual analog scales (VAS) are easy to use and quick to implement. Although the VAS has been validated to assess pain and occupational stress, VAS scores for anxiety and mood have never been evaluated in the workplace. We aimed to validate the use of visual analog scales (VAS) for anxiety and mood compared to HADS in workers. A HADS self-reported questionnaire associated with VAS assessing perceived anxiety and mood on a horizontal line of 100 mm was administered to 182 workers, with a second test (retest) proposed one week later. Sociodemographic, characteristics of work, sleep, well-being, and stress were also assessed. VAS anxiety and mood correlated with the HADS sub-scores (0.70 and 0.65, respectively). The test-retest reliability was good. Optimal VAS cut-offs were ≥ 60/100 for anxiety and ≤ 60/100 for mood, to define at-risk patients. The VAS is quick to perform, easy to use, and reliable for screening depression and anxiety in occupational medicine. We recommend validated questionnaires for at-risk patients.

**Trial registration.** Clinicaltrials.gov: NCT02596737. Available at: https://www.clinicaltrials.gov/ct2/show/NCT02596737.

## Introduction

The World Health Organization (WHO) estimates that mental illness affects one in five people globally and one in three individuals in his/her lifetime [1]. Mental disorders represent the second leading cause of sickness absence [2–4] and the leading cause of disability. Anxiety disorders and mood disorders are common when related to mental illness [5]. The estimated prevalence of anxiety is 18% and mood disorders are 9.5% [6]. Most of them appear at the beginning or during working life [5] and can negatively affect the worker, the organization and the society. Comorbid anxiety and depressive disorders have the greatest impact on long-term work disability and absenteeism, emphasizing the need to address both conditions simultaneously [7]. Psychological ill-health affects human performance and increases the risk of accidents, especially in hazardous industries [8–10]. In manufacturing, an anxious worker is more likely to experience accidents, endangering himself and coworkers, but also increasing downtime and operational costs. Anxiety and depression can lead to both absenteeism [11], and presenteeism [12]. Presenteeism accounts for a significant portion of productivity loss, surpassing direct medical costs for depression [13]. Untreated mental health issues, particularly depression, contribute to a $44 billion annual loss of productive time in the USA [14]. Workplace interventions targeting mental health are moderately effective in improving work outcomes, while programs based on exposure or that combine mental, social and physical health interventions are the most effective [15]. Companies with supportive environments observe a 30% reduction is stress-related absenteeism [16]. For every dollar spent in such programs, companies see up to a $4 return in reduced absenteeism and healthcare costs [17]. Workplace mental health programs and interventions seem to help individual employees but also reduce larger societal costs [17, 18].

Screening and assessing the severity of symptoms is therefore essential to occupational health and should especially happen during consultation by an occupational physician. Among the multiple validated tests assessing anxiety and depression [19–21], the Hospital Anxiety and Depression Scale (HADS) is the most common test. Yet, the questionnaire consists of 14 items and requires extended periods during occupational health consultation, while ooccupational health physicians have limited time to deal with a large number of workers and worksites [22]. Visual analog scales (VAS) are reproducible tools, fast, and easy to use, with good psychometric characteristics, already validated for pain [23], occupational stress [24, 25], and job satisfaction [26]. Therefore, we hypothesized that a VAS for mood and anxiety would identify at-risk workers with depressive or anxious symptoms, in comparison with the HADS.

Stress, sleep disturbance and working hours have been previously been linked with anxiety and depression scores using HADS [27, 28], and should be associated with VAS anxiety and mood.

The main objective of this study was to validate VAS mood and VAS anxiety vs. HADS in a population of active workers. The external validity was evaluated for both VAS by highlighting their relationships with sociodemographic, professional and well-being characteristics.

## Materials and methods

### Participants

An epidemiological, observational, descriptive and cross-sectional study was conducted between 2016-03-01 and 2016-05-12, for which self-questionnaires were sent to users of the WittyFit software [29]. The name WittyFit comes from Witty and Fitness and reflects the concept of health from the World Health Organization (WHO): to be in good physical and mental health. WittyFit is software whose objective is to promote well-being in companies, with an epidemiological and research conception. WittyFit performs a personalized evaluation by self-questionnaire. Participation in the study required written consent. This observational study received approval from the Ethics Committee of the University Hospital of Clermont-Ferrand, France (clinicaltrials.gov identifier NCT02596737) and from National Commission for Information Technology and Civil Liberties (CNIL). All data was anonymous, and the employees’ identity was not collected in the database. A human resource created a number to feed the database, which was then automatically modified by another number.

Participants were invited to participate in the study via the WittyFit software, which delivered the research invite to the entire workforce of participating companies, with the option to refuse. Any worker accepting to join the WittyFit research was considered eligible to participate. No other inclusion criteria were enforced.

### Primary outcome

The primary objective of this study was to validate the two VAS (anxiety and mood) as an alternative to the HADS.

Zigmond and Snaith published HADS for the first time in 1983, in English [30–32]. This was then translated and validated in French by Lépine in 1985 and by Ravazi in 1989 [33–35]. This scale is used for screening for common psychopathological disorders. Seven items evaluate depressive symptoms (items 2, 4, 6, 8, 10, 12, 14): one for dysphoria, one for psychomotor slowdown and five for anhedonia. Seven other items measure anxiety (items 1, 3, 5, 7, 9, 11, 13). Each item is comprised of a four-point Likert scale. The scale of answers is sometimes reversed to avoid repetition bias. The questionnaire generates 2 sub-scores, corresponding to the two subscales of anxiety and depression, with two thresholds set at 7 and 11. Scores 0–7 refer to no anxiety or depressive disorders; 8–10 suspected anxiety or depressive disorders; 11–21 proven anxiety or depressive disorders [30–32, 36].

VAS assessed the perceived anxiety and mood of individuals at work, on a horizontal, non-calibrated line of 100 mm. The scales ranged from very low (0) to very high anxiety (100) for VAS anxiety; and ranging from very sad mood (0) to very good mood (100) for VAS mood.

### Secondary outcomes

Secondary outcomes were defined as the measures of association between the HADS or VAS and workers’ personal and work-related characteristics. Sociodemographic data such as age, gender, education level, and marital status was collected. The characteristics of work were also collected, such as occupation, number of working hours per week, seniority within the company, the type of work schedule (fixed, shift, nightshifts) and the number of nightshifts per month. Body mass index was calculated from height and weight. Sleep quantity was evaluated by the number of sleeping hours per night [37]. Sleep quality, well-being, stress at work, and stress at home were evaluated with VAS ranging from very low (0) to very high (100) [22, 25].

### Time of measurements

Participants could complete the whole questionnaire whenever they wished. All responders were also invited to complete the HADS and VAS anxiety and mood again a week later, to perform the test-retest approach. The handover time was approximately 15 minutes.

### Statistical analysis

Sample size was determined according to COSMIN recommendations [38, 39] as follows. 1) “Rules-of-thumb vary from four to 10 subjects per variable, with a minimum number of 100 subjects to ensure stability of the variance-covariance matrix” and 2) “Often 0.70 is recommended as a minimum standard for reliability. We give a positive rating for reliability when the ICC or weighted Kappa is at least 0.70 in a sample size of at least 50 patients.”

The Stata software (Version 13, StataCorp, College Station, TX) was used, with a a two-sided Type I error of α = 5% to perform statistical analysis. Participants’ characteristics were expressed as mean ± standard deviation (SD) or median [interquartile range] for continuous data (assumption of normality assessed by the Shapiro-Wilk test) and as numbers and associated percentages for categorical parameters.

The test-retest reliability was performed using Pearson correlation coefficient, Lin concordance coefficient and Bland and Altman plots. The external validity was assessed using a correlation coefficient (Pearson or Spearman according to statistical distribution) between VAS and other psychological measures, such as the HADS score. Then, a ROC receiver operating characteristic (ROC) curve analysis were used to determine the optimal thresholds of VAS to predict HADS, according to clinical relevance and usual indexes reported in the literature (Youden index, Liu index and efficiency) [40, 41]. Sensitivity, specificity, positive and negative predictive values were calculated and presented with 95% confidence intervals. The agreement between the HADS anxiety and depression sub-score and corresponding VAS, according to cut-offs determined by ROC curve analysis, was evaluated using agreement rates. Finally, quantitative variables were compared between independent groups by ANOVA or the Kruskal-Wallis test, if ANOVA assumptions were not met (normality and homoscedasticity were analyzed using the Bartlett test). When appropriate, post-hoc tests were performed considering multiple comparisons (Tukey-Kramer post ANOVA and Dunn after Kruskal-Wallis). The comparisons between groups were carried out using the Chi-squared or Fischer’s exact test for categorical variables. When appropriate, a post-hoc test was used (Marascuillo procedure). External validity was assessed by comparing the associations of personal and work-related variables (sex, age, BMI, well-being VAS, sleep quality VAS, sleep duration, stress at work VAS, stress at home VAS, seniority in company, and weekly workload) between participants with and without risks of depression or anxiety, as measured by HADS and VAS. To assess the robustness of our primary analysis, a sensitivity analysis was performed on a subset of the population in which the primary outcomes (VAS anxiety and depression as well as HADS-A and HADS-D), but also the variables used to assess external validity evidence (sex, age, BMI, well-being VAS, Sleep quality VAS, Sleep duration, Stress at work VAS, stress at home VAS, seniority in company, weekly workload) were complete.

## Results

### Participants

**Fig 1:**
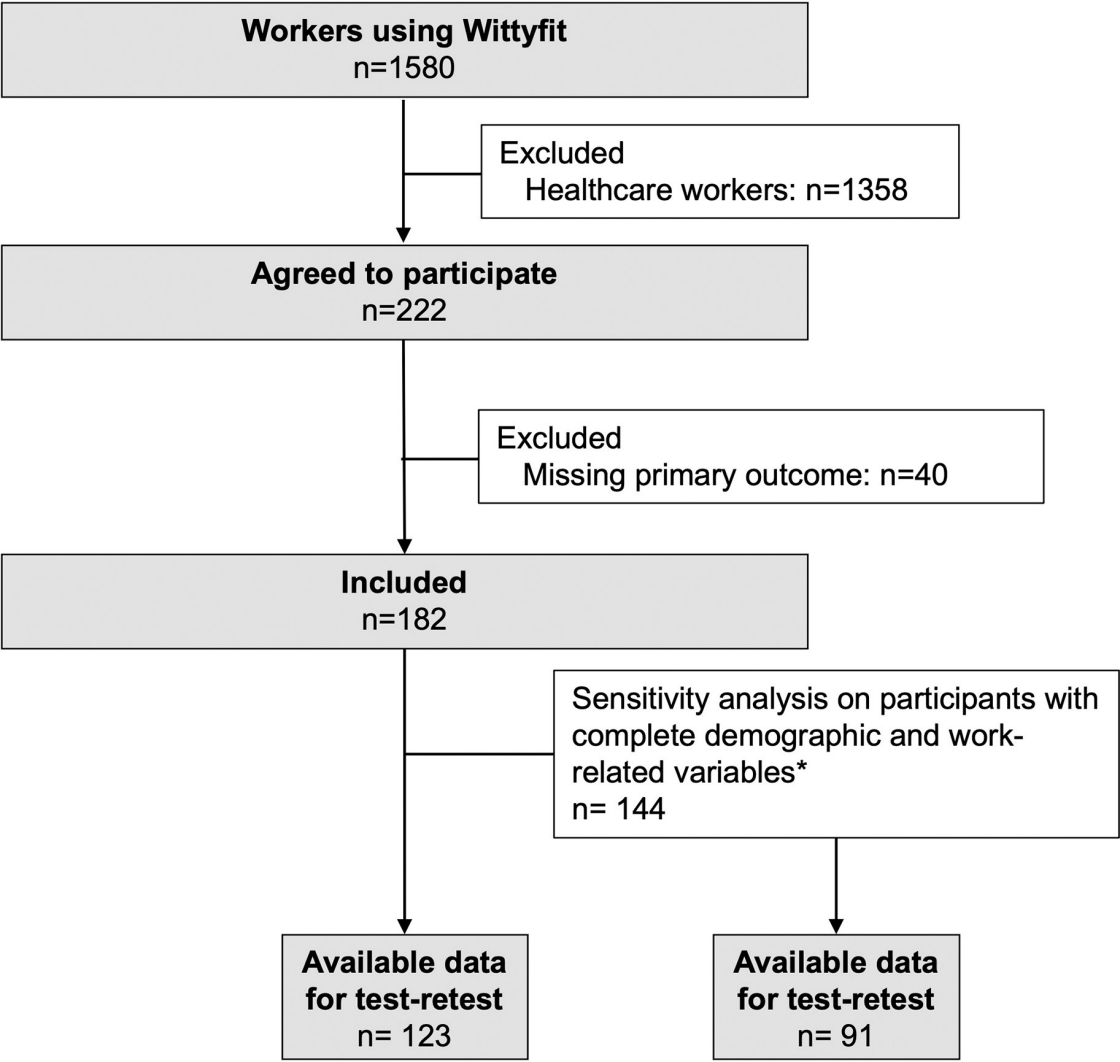
Flowchart and study design. Abbreviations: *: The variables considered to define the sensitivity analysis were sex, age, BMI, well-being VAS, Sleep quality VAS, Sleep duration, Stress at work VAS, stress at home VAS, seniority in company, weekly workload. VAS: Visual Analog Scale.

Among the 1580 workers and users of WittyFit, 222 (14%) agreed to participate. The data for the primary outcomes HADS Anxiety (HADS-A), VAS anxiety, HADS Depression (HADS-D) and VAS mood was missing in 40 participants. The analysis was then conducted on 182 (82%) of the responders. Among them, 86 (47.3%) were women (with 26 missing data). The test-retest approach was calculated on 123 participants who answered twice to the HADS and the two VAS (anxiety and mood) (Fig 1). The average age was 41.4 ± 11.6 years old. Half of the workers were married (44%, 26 missing data). Most included workers were senior executives (57.7%) and had reached a master’s degree or higher (62.6%) (Table 1).

**Table 1:** Characteristics of participants in terms of demographics, education level, marital status, occupational categories and workload.

Characteristics of participants	Sample size (n = 182)	%
**Sex**		
Women	86	47.3
Men	70	38.5
*Missing*	26	14.3
**Age,** years (mean ± standard deviation)	41.4 ± 11.6	
**Education level**		
Undergraduate	10	5.5
Bachelor degree or less	32	17.6
Master degree or more	114	62.6
*Missing*	26	14.3
**Marital status**		
Single	33	18.1
Concubinage	42	23.1
Married	80	44
Widowed	1	0.6
*Missing*	26	14.3
**Occupational categories**		
Senior executives	105	57.7%
Mid level workers	16	8.8%
Skilled workers	26	14.3%
Unemployed	3	1.6%
Retired	6	3.3%
*Missing*	26	14.3%
**Work schedule**		
Fixed	126	69.2%
Daytime Shiftwork	3	1.7%
Shiftwork including nighshift	27	14.8%
*Missing*	26	14.3%
**Number of nighshifts per month**		
0	129	70.9%
1	6	3.3%
2	5	2.7%
3	1	0.5%
4	2	1.1%
5	5	2.7%
6	2	1.1%
7	4	2.2%
8 and more	2	1.1%
*Missing*	26	14.3%
**Number of hours per week**		
Mean ± SD, in hours	41.5 ± 12.1	
0–10 hours	4	2.2%
11–20 hours	5	2.7%
21–30 hours	7	3.8%
31–40 hours	75	41.2%
41–50 hours	38	20.9%
51–60 hours	15	8.2%
61–70 hours	5	2.7%
71–80 hours	1	0.5%
81–90 hours	1	0.5%
*Missing*	31	17%
**Seniority in the company (in years)**		
[0–5[	61	33.5%
[5–10[	30	16.5%
[10–20[	36	19.8%
> = 20	26	14.3%
*Missing*	29	15.9%

### Hospital anxiety and depression scale

To define the threshold on the VAS anxiety and mood compared to the HADS, we assumed for each sub score of anxiety and depression that a score ≤7 was normal and a score ≥ 8 was considered abnormally high.

The mean score of HADS-A was 6.9 ± 4.0. 108 (59.3%) participants had no anxiety symptoms (score ≤7) and 74 (40.7%) had anxiety symptoms (score ≥8) The mean score of HADS-D was 3.8 ± 3.0. 157 (86.3%) participants had no depressive symptoms (score ≤7) and 25 (13.7%) had depressive symptoms (score ≥8) (Table 2).

**Table 2:** Summary statistics for the VAS anxiety, VAS depression, subscale HADS-anxiety, subscale HAD-depression in the study population, segmented according to the chosen cutoff values.

Variables	Sample size n = 182 (%)	Mean ± Standard Deviation
**Visual analog scale (VAS)**		
VAS Anxiety	182	50.4 ± 28.7
<60	106 (58.2%)	
≥60	76 (41.8%)	
VAS Mood	182	65.4 ± 24
≤ 60 (at risk)	67 (36.8%)	
> 60 (not at risk)	115 (63.2%)	
**Hospital Anxiety and Depression Scale (HADS)**		
**HADS Anxiety (HADS-A)**		
HADS-A	182	6.9 ± 4.0
≤ 7	108 (59.3%)	
≥ 8	74 (40.7%)	
**HADS Depression (HADS-D)**		
HADS-D	182	3.8 ± 3.0
≤ 7	157 (86.3%)	
≥ 8	25 (13.7%)	

### VAS anxiety and VAS mood: Cut-offs determination and agreement rate

Mean VAS anxiety was 50.4 ± 28.7. A best cut-off value at 63.5 (*p* <0.001) was calculated to assess the risk of anxiety using VAS Anxiety, with a sensibility of 71.6% (95CI: 59.9% to 81.5%) and a specificity of 86.1% (78.1% to 92.0%), an area under the curve of 0.79 (0.73 to 0.85), a positive predictive value of 78% (66.2% to 87.1%), a negative predictive value of 81.6% (73.2% to 88.2%), and an agreement of 80.2%.

Mean VAS mood was 65.4 ± 24. A best cut-off value at 58 (higher risk for values ≤ 58, *p* <0.001) was calculated to assess the risk of depression using VAS mood, with a sensitivity of 84% (95%CI: 63.9% to 95.5%), a specificity of 72.6% (64.9% to 79.4%), an area under the curve of 0.78 (0.70 to 0.86), a positive predictive value of 32.8% (21.6% to 45.7%) and a negative predictive value of 96.6% (91.5% to 99.1%) and agreement of 74.2%.

**Fig 2:**
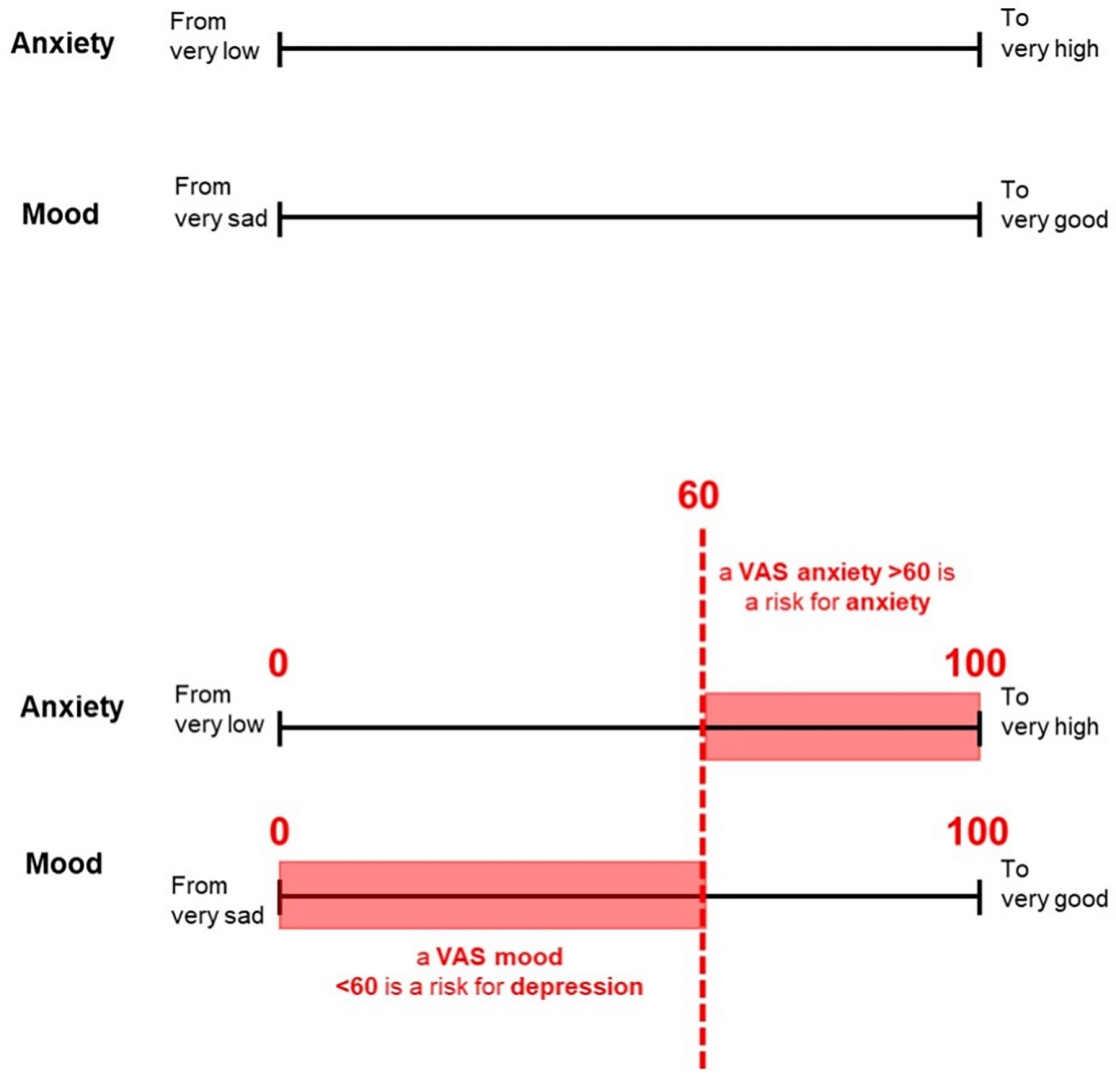
VAS anxiety and mood.

Therefore, we propose to set cut-offs at ≥60 for VAS anxiety and ≤60 for VAS mood. 76 (41.8%) workers had a VAS anxiety ≥60, and 67 (36.8%) workers had a VAS mood ≤ 60 (Table 3, Fig 2).

**Table 3:** Contingency tables of VAS mood and anxiety vs their HADS subscales counterparts, after dichotomization of the VAS around the best cut-off values.

**Mood**				
** **		**VAS Anxiety—Dichotomized**		**Total**
		Normal < 60	At-risk ≥ 60	
**Anxiety subscale of HADS—Dichotomized**	Normal ≤ 7	88 (48.3%)	20 (11%)	**108 (59.3%)**
	At-risk ≥ 8	18 (9.9%)	56 (30.8%)	**74 (40.7%)**
**Total**		**106 (58.2%)**	**76 (41.8%)**	**182**
**Anxiety**				
** **		**VAS Mood—Dichotomized**		**Total**
		Normal > 60	At-risk ≤ 60	
**Depression subscale of HADS—Dichotomized**	Normal ≤7	111 (60.9%)	46 (25.3%)	**157 (86.3%)**
	At-risk ≥8	4 (2.2%)	21 (11.5%)	**25 (13.7%)**
**Total**		**115 (63.2%)**	**67 (36.8%)**	**182**

Using the VAS anxiety, data ranged from minimal to maximal values, with a reasonable standard deviation, as suggested by the variation coefficient around 0.57 for VAS anxiety. Using the VAS mood to 0.30, data ranged from 4 to 100, with a reasonable standard deviation (coefficient of variation of 0.37). No real ceiling of floor effects was observed; with 6% of participants for VAS anxiety and 7.7% for VAS mood having the lowest or highest possible score. The correlation between VAS anxiety and VAS mood was large (Spearman rho 0.51, p < 0.001). VAS anxiety and mood correlated with their respective HADS sub-scores (0.70 and 0.65, respectively, p < 0.001).

### Validation of VAS anxiety and VAS mood—internal validity

#### Correlation between VAS anxiety/VAS mood and HADS-A/HADS-D

Rank correlation coefficient found high correlation (0.70, *p* <0.001) between anxiety score from the HADS-A and the VAS anxiety, and moderate correlation (0.65, *p* <0.001) between depression score from the HADS-D and the VAS mood.

#### Test-retest reproducibility

**Fig 3:**
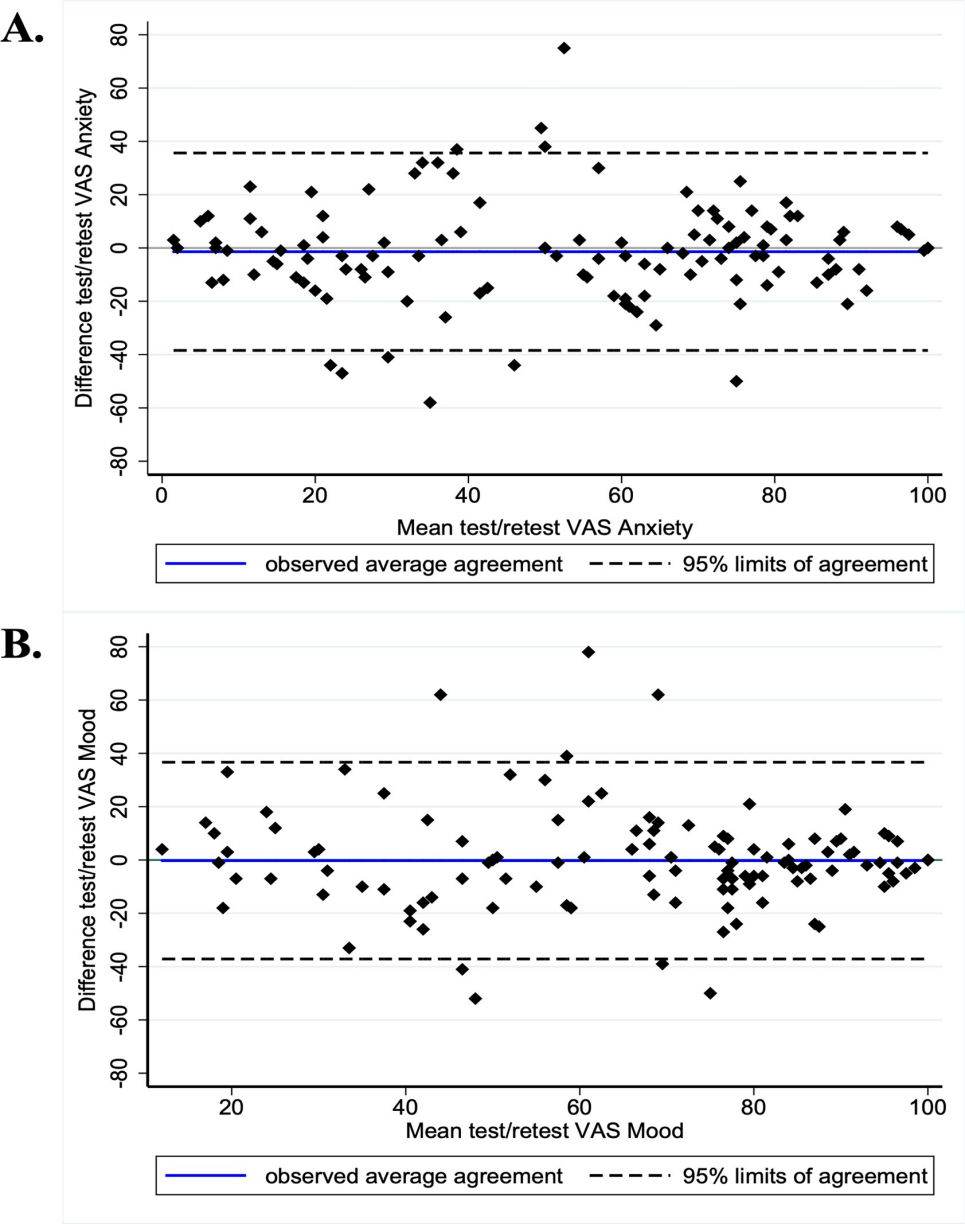
Agreement between the test and retest of both visual analog scales according to Bland-Altman analysis. 2.A. Bland-Altman plot for VAS Anxiety, 2.B. Bland-Altman plot for VAS Mood.

The analysis was performed on a subsample of 123 participants with available data for both test and retest. Lin concordance coefficient for VAS anxiety was 0.79 (95CI: 0.73 to 0.86) with difference on the retest of -1.4 ± 18.9 (95CI: -38.4 to 35.6). Lin concordance coefficient for VAS mood was 0.72 (95CI: 0.64, 0.81) with difference on the retest of -0.22 ± 18.8 (95CI: -37.1 to 36.7). The Bland and Altman plot is shown in Fig 3.

### Validation of VAS anxiety and VAS mood—external validity

#### VAS anxiety

According to the cut-off for VAS anxiety, age, BMI and seniority in the company were not correlated with anxiety symptoms but women were more affected by anxiety than men (*p* = 0.008). Moreover, a high degree of anxiety was correlated with a lower VAS quality of sleep (*p* <0.001) but not with duration of sleep (*p* = 0.46). Also, a high degree of anxiety was associated with higher stress at work (*p* <0.001) and at home (*p* <0.001) but not with a longer time spent at work (*p* = 0.7). Conversely, a lower VAS anxiety was linked to higher VAS well-being (*p* <0.001). Relationships were similar using the HADS-A (Table 4).

**Table 4:** Association between anxiety and depression constructs (VAS or HADS subscales) and participants characteristics (demographics, well-being, sleep, stress and work-related characteristics). The agreement between VAS and HADS subscale is indicated for each characteristic. Abbreviations: Mean ± SD: Mean ± Standard deviation. Statistically significant results are displayed in bold.

Variables		Anxiety				Depression			
		Visual analog scale		HAD-A of HADS		Visual analog scale		HAD-D of HADS	
		< 60	≥ 60	≤ 7	≥ 8	≥ 60	< 60	≤ 7	≥ 8
**Age**									
** **	Mean ± SD	42.7 ± 11.6	39.7 ±11.5	42.3 ± 11.9	40.4 ± 11.1	42.1 ± 11.5	38.6 ± 11.8	41.5 ± 11.8	41.1 ± 10.8
** **	p-value	0.12		0.34		0.15		0.94	
** **	Agreement	Yes				Yes			
**Sex**									
** **	Women (n) / Men (n)	42 / 49	44 / 21	42 / 49	44 / 21	68 / 60	18 / 10	73 / 63	13 / 7
** **	p-value	**0.008**		**0.008**		0.3		0.34	
** **	Agreement	Yes				Yes			
**Body Mass Index**									
** **	Mean ± SD	24.6 ± 4.8	23.5 ± 3.9	24.5 ± 4.3	23.8 ± 4.6	24.1 ± 4.4	24.7 ± 4.9	23.9 ± 4.2	25.7 ± 6
** **	p-value	0.097		0.09		0.76		0.27	
** **	Agreement	Yes				Yes			
**VAS Well-being**									
** **	Mean ± SD	67.9 ± 20.1	53.2 ± 20.5	68.9 ± 19.6	51.6 ± 20	67.4 ± 17.9	34.8 ± 16	65.4 ± 19	38.5 ± 22
** **	p-value	**<0.001**		**<0.001**		**<0.001**		**<0.001**	
** **	Agreement	Yes				Yes			
**VAS Quality of sleep**									
** **	Mean ± SD	62.7 ± 27	45.5 ± 24.9	63 ± 26.8	44.9 ± 24.9	60.6 ± 26.5	31.1 ± 16.7	58.5 ± 26.8	35.8 ± 23.8
** **	p-value	**<0.001**		**<0.001**		**<0.001**		**<0.001**	
** **	Agreement	Yes				Yes			
**Duration of sleep**									
** **	Mean ± SD	427.8 ± 52.3	422.4 ± 62.1	425.3 ± 55.5	423.8 ± 58.5	428.1 ± 55.9	413.2 ± 59.4	427.74 ± 56.5	411.2 ± 56.3
** **	p-value	0.46		0.83		0.2		0.14	
** **	Agreement	Yes				Yes			
**VAS Stress at home**									
** **	Mean ± SD	28.9 ± 24.4	46.4 ± 22.2	29.7 ± 24.8	45.7 ± 22.1	34.6 ± 25	44.3 ± 23.4	34 ± 24.3	50.9 ± 24.5
** **	p-value	**<0.001**		**<0.001**		**0.04**		**0.002**	
** **	Agreement	Yes				Yes			
**VAS Stress at work**									
** **	Mean ± SD	49.5 ± 23.6	67.8 ± 20.5	49.2 ± 23.1	68.6 ± 20.6	54.6 ± 22.6	69.6 ± 27.1	55.6 ± 23.7	67.5 ± 24.1
** **	p-value	**<0.001**		**<0.001**		**<0.001**		**0.01**	
** **	Agreement	Yes				Yes			
**Seniority in company**									
** **	Mean ± SD	11.8 ± 11.1	9.4 ± 9.5	11.5 ± 10.6	9.7 ± 10.4	11.4 ± 10.9	7.8 ± 8	11.1 ± 10.8	8.3 ± 8.2
** **	p-value	0.29		0.22		0.07		0.4	
** **	Agreement	Yes				Yes			
**Number of hours of work per week**									
** **	Mean ± SD	42 ± 11	40.8 ± 13.5	41.3 ± 12.4	41.8 ± 11.8	42.3 ± 11.8	37.9 ± 13	42.2 ± 11.9	37.2 ± 12.9
** **	p-value	0.7		0.63		0.18		0.09	
** **	Agreement	Yes				Yes			

#### VAS mood

According to the cut-off for VAS mood, age, sex, BMI and seniority in the company were not correlated with anxiety symptoms. A sad mood was associated with a lower VAS quality of sleep (*p* <0.001) but not with duration of sleep (*p* = 0.2). Also, sad mood was associated with higher stress at work (*p* <0.001) and at home (*p* = 0.04) but not with longer time spent at work (*p* = 0.18). Conversely, good mood was linked to better VAS well-being (*p* <0.001). Relationships were similar using the HADS-D (Table 4).

#### Sensitivity analysis

A sensitivity analysis was performed on the group of 144 patients for which complete data were available regarding the primary outcome and defining variables (sex, age, BMI, well-being VAS, Sleep quality VAS, Sleep duration, Stress at work VAS, stress at home VAS, seniority in company, weekly workload), presented in S1 Table. The distributions of HADS-A and HADS-D in this subset was similar to those observed in the entire cohort. The mean score of HADS-A was 6.9 ± 3.9. 83 (57.6%) participants had no anxiety symptoms (score ≤7) and 61 (42.4%) had anxiety symptoms (score ≥8) The mean score of HADS-D was 3.8 ± 2.96. 126 (87.5%) participants had no depressive symptoms (score ≤7) and 18 (12.5%) had depressive symptoms (score ≥8). Regarding VAS anxiety, the same optimal cutoff of 63.5 was drawn from this sample and yielded similar classification performance. The analysis led of VAS mood led to the same optimal cutoff of 42 and yielded a similar classification performance.

The analysis of test-retest reproducibility was performed on a subsample of 91 participants with available data for both test and retest and yielded similar results compared to those observed in the entire cohort. Lin concordance coefficient for VAS anxiety was 0.79 (95CI: 0.71 to 0.87) with difference on the retest of -1.37 ± 19.5 (95CI: -39.6 to 36.8). Lin concordance coefficient for VAS mood was 0.75 (95CI: 0.65, 0.84) with difference on the retest of -0.42 ± 18.7 (95CI: -36.9 to 36.1). The Bland-Altman analysis is shown in S1 Fig.

Evidence of external validity were similar in this subset compared to the entire cohort and concordant between the VAS and the HADS assessment for both anxiety and depression. The only discrepancy was observed for VAS stress at work, that was not significantly different between the groups without vs. with suspected or proven depressive disorder (HADS-D ≥ 8) (56.3 ± 22.7 vs. 61.8 ± 25.1, respectively, p = 0.33, Cohen’s d = 0.55), while the groups at-risk of depressive disorder according to the VAS Mood exhibited a significantly higher weekly workload compared to the group not at risk (62.5 ± 24.0 vs. 53.5 ± 21.8, respectively, p = 0.01, Cohen’s d = 0.33). Those results are presented in S2 Table.

## Discussion

This study brings validity evidence of visual analog scales of anxiety and mood for clinical use in occupational health, i.e., the assessment of its acceptability, reproducibility, internal and external validity.

### Prevalence and health outcomes

Anxiety and mood disorders are common illnesses in active or non-active populations [1, 42–50]. In our study, 41.8% and 36.8% of workers reported anxiety (VAS anxiety ≥ 60/100) and depressive (VAS mood ≤ 60/100) symptoms. The literature confirms that rates are variable, ranging from 12 to 73% depending on the profession, the country of study, and the questionnaire used [46–48, 50–53]. Those disorders increase the risk of numerous diseases [54], such as metabolic and cardiovascular diseases [55, 56]. Anxiety and depression are also major risk factors for suicide [57, 58]. Psychological disorders are not correctly detected or diagnosed [59–61], and this can significantly impact patients’ professional life such as discrimination in hiring, stigmatization in the workplace, difficulty in maintaining employment, and absenteeism [2–4, 49, 62, 63]. More than half of employers would never accept someone diagnosed with depression for a managerial position, and more than one third of workers would be anxious about such colleagues [64, 65]. Conversely, occupational factors can directly promote anxious-depressive symptomatology such as stress [47, 53, 66–68], damaging social relationships [67] and management [63], imbalanced job design [69], occupational uncertainty, or lack of value and respect in the workplace [66]. The occupational physicians hold an important role in promoting mental health through individual and collective prevention [70], early identification of mental pathologies, and maintenance during return to work [71, 72].

### Acceptability

Ceiling or floor effects happen when 15% of participants have the highest or lowest possible score, indicating a lack of discrepancy for extreme scores [73]. No real ceiling of floor effects were observed; with less than 15% of participants having the lowest or highest possible score, as recommended in the literature [73]. The absence of major floor and ceiling effect demonstrate the absence of over-representation of extreme levels of mood and anxiety, and therefore the pertinence of VAS to discriminate symptoms of anxiety or depression [39, 74]. We retrieve one study mentioning ceiling effects in the group of healthy subjects regarding the use of electronic models of VAS pain, anxiety, fatigue and quality of life [75]. However, most of the available studies did not systematically stipulate floor or ceiling effects [23–26, 76–80]. Only one study evaluated the floor and ceiling effects for HADS and found a floor effect for each item but no ceiling effect. However, this study did not concern active subjects but the general population aged 65 to 80 [81]. Despite the lack of data on the time required to complete the VAS tools and the HADS, we assume that there must be a benefit in completion times, as it takes a few seconds to answer a VAS, while HADS takes up to 5–10 minutes [82–84].

### Internal validity

In our study, the agreement and reproducibility of VAS anxiety and mood measured by Lin concordance coefficients were satisfying and were over 0.70 for the two VAS. The best sensitivity/specificity compromises were for a threshold of 60/100 for each VAS anxiety and VAS mood: at-risk workers being those for VAS anxiety ≥ 60/100 and those for VAS mood ≤ 60/100. The reliability, the validity and the sensibility of VAS anxiety and VAS mood were also confirmed in the literature versus other questionnaires than HADS, and within specific populations (preanesthesia, dental care for anxiety [76–78, 85], after a stroke or in geriatrics for depression and mood [86–90]. Moreover, VAS anxiety was moderately correlated with VAS mood (coefficient = 0.51; p <0.001), aligned with the literature reporting similar correlations between the HADS-A and the HADS-D (varying from 0.40 to 0.74, mean 0.56) [91–93]. The visual graphical analysis of Bland and Altman for both VAS showed no systematic error but a relatively large random error with a heterogeneous general dispersion; the 95% limits were adequate for high and low values of mean VAS anxiety and mean VAS mood test and retest. We have found similar data in the literature for VAS pain, fatigue, quality of life and anxiety, in a population with multiple sclerosis [75]. The test/retest differences were acceptable [94, 95].

### External validity

This study demonstrated a very good external validity of VAS anxiety/VAS mood: the same relationships were shown between those VAS and other variables, and between HADS-A / HADS-D and those same variables. Women had more anxiety symptoms (both for VAS anxiety and HADS-A) without differences for depressive symptoms (both for VAS mood and HADS-D). The literature also frequently report higher levels of anxiety in women, with more heterogeneous data for mood [32, 48–51, 92]. Whatever the scales considered (VAS anxiety vs. HADS-A, or VAS mood vs. HADS-D), age did not influence depressive and anxiety symptoms. The influence of age on those symptoms is conflicting in the literature, depending on the population studied [28, 48, 53, 92]. Similarly, whatever the scales considered, anxiety and depressive symptoms were associated with a poor sleep quality and high levels of stress at work or at home. In line with the literature, anxiety and mood perturbations may be associated with a poor sleep [48] and work- or home-related stress [46, 96]. In our study, other factors (such as working hours and seniority in the company) were not associated with anxiety and mood. The literature did not have consensus data on the protective [50] or vulnerability [48] effects associated with seniority in the company. However, a positive correlation was found between working hours, anxiety and depression [28]. However, despite no significance, relationships were similar between VAS and HADS, and those factors.

### Limitations

Compared to other French studies using a questionnaire, the response rate may seem low [29, 97–101]. However, the number of included workers made it possible to carry out the statistical analyses with the number of required subjects, determined *a priori [38, 39]*. Also, we had some missing data despite the volume of the survey [102]. Moreover, the proportion of participants who answered both test and retest was higher in our study than in other studies [83], demonstrating the interest of workers for questionnaires on quality of life at work. A limitation arose from participants’ characteristics with more women than men, and a higher representation of executives (57%) compared to mid-level professionals (23%). This class imbalance in terms of socio-economic levels calls for larger studies designed to assess the validity evidence of these VAS in other socio-economic classes. Considering internal validity, our study did not assess sensitivity to changes in anxiety and mood. However, test-retest reliability was acceptable. Moreover, literature previously showed a good sensitivity to changes using VAS anxiety before and after stress [76, 95]. The relatively large random error retrieved at the Bland and Altman test may traduce the non-negligible probability of a stressful event having occurred between the test and retest. Also, we suggest that the same evaluation may be repeated for people close to the at-risk threshold, which is relatively easy considering the instantaneous time required to answer. Our study may suffer from limitations in terms of external validity because we used only the HADS whereas other validated assessment tools exist to assess anxiety and depressive symptoms. However, HADS is classically considered as the gold standard with good external structure and satisfying discriminant validity [103]. Moreover, the aim of our study was not to compare the numerous scales but rather to validate the VAS anxiety and mood vs. the reference test, in a general population. We also used many sociodemographic, occupational, and clinical data for external validity. Despite a sufficient sample size for the validation of VAS anxiety and mood, insufficient data precluded further analyses on at-risk occupations for anxiety and depressive symptoms. However, all professions can be affected by the common mental disorders of anxiety and depression due to major work changes. Indeed, some studies showed that intellectual professions or higher degrees have high levels of anxiety and depression [48, 50], while others reported a high risk for lowest occupational categories (workers and technical classes) [92, 104]. Identifying at-risk workers is necessary for effective preventive strategies. Assessment of anxiety by a VAS was only described in pre-anesthesia patients [77, 78] or dental care, limiting its generalizability and its validity, as it was not compared with the HADS. Finally, considering semantics, VAS mood presented in the literature had a specific design. They used a combination of words and schematic faces indicating different mood states. Those VAS were designed for certain profiles of patients with neurologic disorders or cognitive impairment [79, 80, 86, 88]. Therefore, we were the first to introduce a unique VAS anxiety and mood model in general population.

## Conclusion

Our results show that VAS anxiety and VAS mood are reliable tools for identifying at-risk workers for anxiety and depression, allowing for a quicker screening and primary prevention in the workplace. We have determined a cut-off value of 60 for each of the VAS: ≥ 60/100 for workers at risk of anxiety (Sensitivity 72%, Specificity 86%, ROC AUC 0.79) and ≤ 60/100 for depression (Sensitivity 84%, Specificity 73%, ROC AUC 0.78). The Anxiety and Mood VAS yielded satisfying test-retest reliability and their association with participants socio-demographic and work-related characteristics are similar with those observed for the HADS.

## Supporting information

S1 Table Characteristics of participants in the sensitivity analysis, defined by the subgroup in which the primary outcome and the variables used for examining external validity evidence were complete. Those variables are sex, age, BMI, well-being VAS, Sleep quality VAS, Sleep duration, Stress at work VAS, stress at home VAS, seniority in company, weekly workload).
(DOCX)

S2 Table Agreement between visual analog scales vs. HADS on differences in participants’ characteristics in the sensitivity analysis cohort. Abbreviations: Mean ± SD: Mean ± Standard deviation. Statistically significant results are displayed in bold.
(DOCX)

S1 Fig Agreement between the test and retest of both visual analog scales according to Bland-Altman analysis, in the sensitivity analysis cohort. A. Bland-Altman plot for VAS Anxiety, B. Bland-Altman plot for VAS Mood.
(DOCX)
