# Supplementary material for: Validation of visual analog scales of mood and anxiety at the workplace
Source: PLoS One. 2024 Dec 31;19(12):e0316159. doi: 10.1371/journal.pone.0316159 (PMC11687878; doi:10.1371/journal.pone.0316159)
Supplement: S1 Table — Those variables are sex, age, BMI, well-being VAS, Sleep quality VAS, Sleep duration, Stress at work VAS, stress at home VAS, seniority in company, weekly workload). (DOCX) [file pone.0316159.s001.docx]

**S1 Table: Characteristics of participants in the sensitivity analysis, defined by the subgroup in which the primary outcome and the variables used for examining external validity evidence were complete.** Those variables are sex, age, BMI, well-being VAS, Sleep quality VAS, Sleep duration, Stress at work VAS, stress at home VAS, seniority in company, weekly workload)

| **Characteristics of participants** | **Sample size (n=144)** | **%** |
| --- | --- | --- |
| **Sex** |  |  |
| Women | 79 | 54.9% |
| Men | 65 | 45.1% |
| **Age,** years (mean±standard deviation) | 41.1 ± 10.9 | |
| **Education level** |  |  |
| Bachelor degree or less | 5 | 3.5% |
| Undergraduate | 28 | 19.4% |
| Master degree or more | 111 | 77.1% |
| **Marital status** |  |  |
| Single | 27 | 18.8% |
| Concubinage | 40 | 27.8% |
| Married | 76 | 52.8% |
| Widowed | 1 | 0.7% |
| **Occupational categories** |  |  |
| Senior executives | 103 | 71.5% |
| Mid level workers | 13 | 9.0% |
| Skilled workers | 23 | 16.0% |
| Unemployed | 2 | 1.4% |
| Retired | 3 | 2.1% |
