# Supplementary material for: Validation of visual analog scales of mood and anxiety at the workplace
Source: PLoS One. 2024 Dec 31;19(12):e0316159. doi: 10.1371/journal.pone.0316159 (PMC11687878; doi:10.1371/journal.pone.0316159)
Supplement: S2 Table — Abbreviations: Mean ± SD: Mean ± Standard deviation. Statistically significant results are displayed in bold. (DOCX) [file pone.0316159.s002.docx]

### **S3 Table: Agreement between visual analog scales vs. HADS on differences in participants’ characteristics in the sensitivity analysis cohort.** Abbreviations: Mean ± SD: Mean ± Standard deviation. Statistically significant results are displayed in bold.

| **Variables** | | **Anxiety** | | | | **Depression** | | | |
| --- | --- | --- | --- | --- | --- | --- | --- | --- | --- |
|  |  | **Visual analog scale** | | **HAD-A of HADS** | | **Visual analog scale** | | **HAD-D of HADS** | |
|  |  | < 60 | ≥ 60 | ≤ 7 | ≥ 8 | ≥ 60 | < 60 | ≤ 7 | ≥ 8 |
| **Age** |  |  |  |  |  |  |  |  |  |
|  | Mean ± SD | 41.8 ± 10.9 | 40 ± 11.0 | 41.8 ± 11.3 | 40.0 ± 10.3 | 41.1 ± 11.2 | 40.9 ± 10.6 | 40.8 ± 11.1 | 43.1 ± 9.4 |
|  | p-value | 0.34 | | 0.38 | | 0.93 | | 0.29 | |
|  | Agreement | Yes | | | | Yes | | | |
| **Sex** |  |  |  |  |  |  |  |  |  |
|  | Women (n) / Men (n) | 36 / 47 | 43 / 18 | 37 / 46 | 42 / 19 | 43 / 45 | 36 / 20 | 67 / 59 | 12 / 6 |
|  | p-value | **<0.001** | | **0.003** | | 0.09 | | 0.2 | |
|  | Agreement | Yes | | | | Yes | | | |
| **Body Mass Index** | |  |  |  |  |  |  |  |  |
|  | Mean ± SD | 24.7 ± 4.9 | 23.5 ± 3.9 | 24.6 ± 4.4 | 23.7 ± 4.7 | 24.3 ± 4.4 | 24.1 ± 4.6 | 23.0 ± 4.2 | 25.8 ± 6.1 |
|  | p-value | 0.10 | | 0.06 | | 0.58 | | 0.24 | |
|  | Agreement | Yes | | | | Yes | | | |
| **VAS Well-being** |  |  |  |  |  |  |  |  |  |
|  | Mean ± SD | 67.5 ± 20.5 | 53.0 ± 19.5 | 68.2 ± 19.7 | 59.0 ± 19.8 | 72.1 ± 15.1 | 44.4 ± 18.2 | 60.1 ± 18.6 | 35.3 ± 20.8 |
|  | p-value | **<0.001** | | **<0.001** | | **<0.001** | | **<0.001** | |
|  | Agreement | Yes | | | | Yes | | | |
| **VAS Quality of sleep** | |  |  |  |  |  |  |  |  |
|  | Mean ± SD | 65.7 ± 26.4 | 46.0 ± 24.7 | 65.7 ± 26.0 | 45.9 ± 25.0 | 66.2 ± 25.4 | 43.3 ± 24.5 | 60.5 ± 34.7 | 34.7 ± 22.8 |
|  | p-value | **<0.001** | | **<0.001** | | **<0.001** | | **<0.001** | |
|  | Agreement | Yes | | | | Yes | | | |
| **Duration of sleep** | |  |  |  |  |  |  |  |  |
|  | Mean ± SD | 428.5 ± 49.4 | 421.5 ± 60.6 | 425.2 ± 54.6 | 425.9 ± 54.4 | 427.7 ± 54.8 | 422.1 ± 53.9 | 426.5 ± 54.0 | 418.3 ± 57.7 |
|  | p-value | 0.40 | | 0.83 | | 0.57 | | 0.35 | |
|  | Agreement | Yes | | | | Yes | | | |
| **VAS Stress at home** | |  |  |  |  |  |  |  |  |
|  | Mean ± SD | 28.1 ± 23.2 | 46.3 ± 22.4 | 29.0 ± 24.5 | 45.1 ± 21.5 | 29.3 ± 24.8 | 45.9 ± 20.4 | 34.2 ± 24.0 | 46.7 ± 25.5 |
|  | p-value | **<0.001** | | **<0.001** | | **<0.001** | | **0.02** | |
|  | Agreement | Yes | | | | Yes | | | |
| **VAS Stress at work** | |  |  |  |  |  |  |  |  |
|  | Mean ± SD | 50.3 ± 22.7 | 66.1 + 20.3 | 49.7 ± 22.4 | 66.9 ± 20.2 | 53.5 ± 21.8 | 62.5 ± 24.0 | 56.3 ± 22.7 | 61.8 ± 25.1 |
|  | p-value | **<0.001** | | **<0.001** | | **0.01** | | 0.33 | |
|  | Agreement | Yes | | | | No | | | |
| **Seniority in company** | |  |  |  |  |  |  |  |  |
|  | Mean ± SD | 10.9 ± 10.3 | 9.8 ± 9.6 | 11.4 ± 10.6 | 9.0 ± 8.9 | 10.4 ± 10.4 | 10.3 ± 9.3 | 10.6 ± 10.2 | 9.1 ± 8.3 |
|  | p-value | 0.75 | | 0.18 | | 0.76 | | 0.85 | |
|  | Agreement | Yes | | | | Yes | | | |
| **Number of hours of work per week** | |  |  |  |  |  |  |  |  |
|  | Mean ± SD | 42.7 ± 10.4 | 41.5 ± 13.3 | 42.5 ± 11.7 | 41.8 ± 11.8 | 43.4 ± 11.6 | 40.2 ± 11.7 | 42.8 ± 11.4 | 38.1 ± 12.9 |
|  | p-value | 0.71 | | 0.97 | | 0.22 | | 0.12 | |
|  | Agreement | Yes | | | | Yes | | | |
