# Supplementary material for: Validation of visual analog scales of mood and anxiety at the workplace
Source: PLoS One. 2024 Dec 31;19(12):e0316159. doi: 10.1371/journal.pone.0316159 (PMC11687878; doi:10.1371/journal.pone.0316159)

**S2 Figure: Agreement between the test and retest of both visual analog scales according to Bland-Altman analysis, in the sensitivity analysis cohort.** A. Bland-Altman plot for VAS Anxiety, B. Bland-Altman plot for VAS Mood.


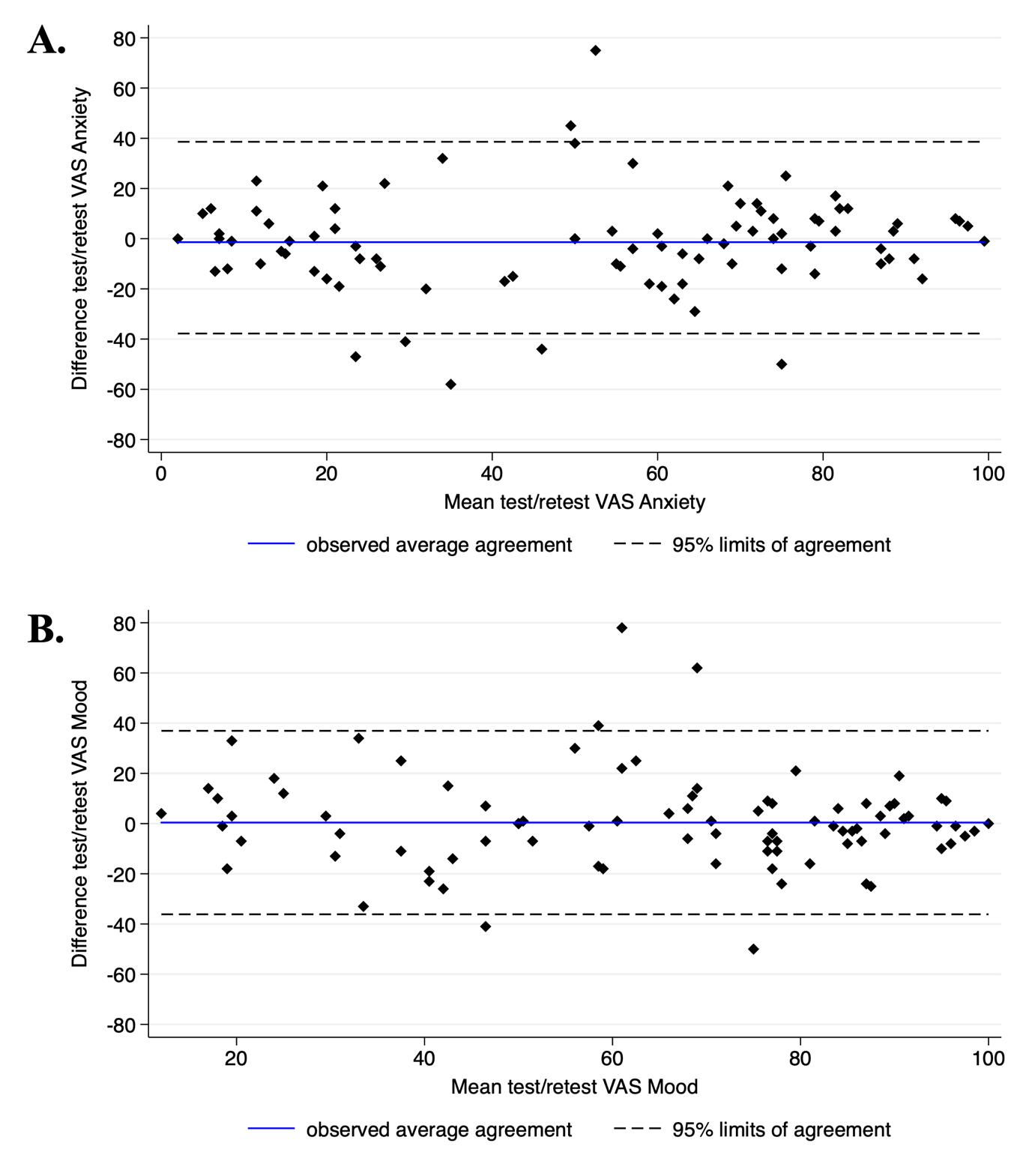

Supplement: S1 Fig — A. Bland-Altman plot for VAS Anxiety, B. Bland-Altman plot for VAS Mood. (DOCX) [file pone.0316159.s003.docx]
